# Targeting Metabolism as a Platform for Inducing Allograft Tolerance in the Absence of Long-Term Immunosuppression

**DOI:** 10.3389/fimmu.2020.00572

**Published:** 2020-04-09

**Authors:** Chih-Hsien Cheng, Chen-Fang Lee, Byoung Chol Oh, Georg J. Furtmüller, Chirag H. Patel, Gerald Brandacher, Jonathan D. Powell

**Affiliations:** ^1^Sidney∼Kimmel Comprehensive Cancer Research Center, Department of Oncology, Johns Hopkins University School of Medicine, Baltimore, MD, United States; ^2^Bloomberg∼Kimmel Institute for Cancer Immunotherapy, Johns Hopkins University School of Medicine, Baltimore, MD, United States; ^3^Department of Liver and Transplantation Surgery, Chang-Gung Memorial Hospital, Chang-Gung Transplantation Institute, Chang-Gung University College of Medicine, Taoyuan, Taiwan; ^4^Vascularized Composite Allotransplantation Laboratory, Department of Plastic and Reconstructive Surgery, Johns Hopkins University School of Medicine, Baltimore, MD, United States

**Keywords:** mouse, model, transplantation, immunology, metabolism, costimulation blockade, rejection

## Abstract

Transplant tolerance in the absence of long-term immunosuppression has been an elusive goal for solid organ transplantation. Recently, it has become clear that metabolic reprogramming plays a critical role in promoting T cell activation, differentiation, and function. Targeting metabolism can preferentially inhibit T cell effector generation while simultaneously promoting the generation of T regulatory cells. We hypothesized that costimulatory blockade with CTLA4Ig in combination with targeting T cell metabolism might provide a novel platform to promote the induction of transplant tolerance.

## Introduction

Transplantation is now recognized as the most effective therapy for patients with end stage organ failure. Despite outstanding short-term graft and patient survival, organ transplantation continues to face several major challenges including poor long-term graft survivadyl resulting from chronic rejection (1–4) and major side effects from the need for long-term immunosuppressive therapy (5, 6). Along these lines, a long elusive goal of human organ transplantation has been the development of therapeutic prophylaxis to prevent graft rejection that ultimately induce transplant tolerance in the absence of long-term immunosuppression.

To this end, maintenance of immunosuppression and treatments for graft rejection rely heavily on the use of calcineurin inhibitors (CNIs) (7–10). These regimens consist of truly potent immunosuppressive agents in that not only do they inhibit immune activation but they also inhibit the induction of tolerance (11, 12). Indeed T regulatory cell generation, activation induced cell death, and T cell anergy are all inhibited by calcineurin inhibitors (13, 14). More recently, the use of costimulatory blockade has been employed in experimental and clinical transplant protocols. T cell activation require signals elicited by the T cell receptor (TCR), costimulatory receptors and the immune microenvironment (15, 16). CD28 is expressed on the surface of the majority of naïve CD4+ and CD8 T+ cells and is the major costimulatory molecule in initial T cell activation (17). Targeting CD28/B7 T-cell co-stimulation pathways with CTLA4Ig to reduce pathological T-cell responses has met with therapeutic success in transplantation, but challenges remain (18). Data from phase III clinical trials have shown promising results with significant improvement in risk of death, graft loss, donor-specific antibodies and better graft function with CTLA4Ig compared to CNIs (19, 20). However, regimens with CTLA4Ig have also shown higher incidence and severity of acute rejection, especially during the early phase post-transplantation (21, 22). Furthermore, while costimulatory blockade has been shown to promote tolerance in animal models of transplantation, such has not been achieved with these agents in human trials (23, 24).

It is becoming increasingly clear that immune cell activation, differentiation, and function is intimately linked to cellular metabolic reprogramming (25, 26). Similar to cancer cells, activated T cells markedly upregulate glycolysis even in the presence of oxygen. Simultaneously, metabolites generated through the tricarboxylic acid cycle (TCA cycle) can be employed to generate the substrates (amino acids, lipids and nucleic acids) for the prodigious anabolic demands of activation. To this end, glutamine plays a critical role in promoting this process through its conversion to glutamate and subsequently alpha ketoglutarate (27–30). While these metabolic processes are critical for T cell effector function, naïve T cells and regulatory T cells rely on more conventional metabolic programs such as oxidative phosphorylation and fatty acid oxidation. With this in mind, we recently demonstrated the ability of anti-metabolic therapy to prevent graft rejection in mouse models of transplantation (31). Using this approach we were able to maximally inhibit the expansion and function of antigen specific effector cells while promoting the generation of antigen specific regulatory T cells. Nonetheless, long term allograft survival in heart transplants required continued drug treatment. That is, while our metabolic therapy inhibited effector function and promoted regulatory T cells, this was not enough to promote allograft tolerance in the absence of treatment.

In this study, we investigated the effect of combining CTLA4Ig abatacept with our previously defined metabolic inhibitor (MI) therapy. This regimen consists of the glucose analog 2DG which blocks glycolysis, the glutamine analog 6-Diazo-5-oxo-L-norleucine (DON) and the diabetes drug metformin (which blocks complex I of the mitochondria). Our data demonstrate that the addition of CTLA4Ig to continuous metabolic therapy not only results in enhanced skin allograft survival but also promotes long-term cardiac allograft acceptance in the absence of maintenance treatment.

## Methods

### Mice

C57BL/6 (H-2b), BALB/c (H-2d), FVB/N (H2q), FVB-Tg(CAG-luc,-GFP)L2G85Chco/J (H2q), B6(Cg)-Tyrc-2J/J (H2b) mice were purchased from The Jackson Laboratory. 5C.C7 mice were purchased from Taconic Farms. All animal procedures were in accordance with the guidelines of the Institutional Animal Care and Use Committee (IACUC) at Johns Hopkins University.

### Antibodies and Reagents

Antibodies against the following proteins were purchased from eBioscience: CD44 (IM7, 1:500), IFN gamma (XMG1,2, 1:500), T-bet (4B10, 1:500), Ki-67 (501A15, 1:1000), KLRG1 (2F1, 1:500), Foxp3 (FJK-16S, 1:250), B220 (RA3-6B2, 1:500), IgG and Fc Block (2.4G2, 1:100). Antibodies against CD3 (145-2C11, 1:1000), Bcl6(K112-91, 1:200), CD4 (RM4-5, 1:1000), CD8 (53-6,7, 1:1000), IFN gamma (XM61,2 1:1000), CD25 (PC61, 1:1000) were purchased from BD Biosciences. Annexin V (BD Bioscience) staining was performed to manufacturer's protocol using Annexin V buffer. Antibodies against granzyme B (GB11, 1:500), PD1 (29F1.A12, 1:500) were purchased from Biolegend. The following antibodies were purchased from Cell Signaling: p-S6 (S240/244, D68F8, 1:1000), phospho PLC gamma (Tyr 783, 1:2000 for immunoblotting). OVA-I peptide (SIINFEKL) was purchased from AnaSpec. Stimulatory anti-CD3 (2C11) and anti-CD28 (37.51) were purified from hybridoma supernatants prepared “in-house.” CFSE was obtained from Invitrogen. Cell Proliferation Dye-eFluor450 and fixable viability dye eFluor780 were purchased from eBiosciences. PMA, and ionomycin were purchased from Sigma Aldrich. Class I OVA peptide was obtained from AnaSpec. Vaccinia-OVA (1E6 pfu) and listeria-OVA (5E6 cfu) are modified vaccinia and listeria that contain the full-length ovalbumin protein but lack lytic ability and were generated as previously described (32).

### OTI CD8+ T Cell Adoptive Transfer

Naïve Thy1.1 OTI CD8+ T cells were labeled with eF-450 cell proliferation dye. 1-2.5 × 10^6^ cells CD8+ T cells were transferred into naïve C57/Bl6J (Thy1.2) hosts already infected with vaccinia-OVA (4 h prior). Mice were subsequently treated with same drug regimen for transplantation but received DON for both days rather than every other day. On Day 2, spleens were harvested and donor T cells (Thy1.1) were analyzed for cell proliferation and cell death.

### Cell Culture

Splenocytes or T cells were cultured in RPMI 1640 media supplemented with 10% FBS, penicillin/streptomycin, glutamine and 50 μM BME. For naïve stimulation and proliferation studies, splenocytes from C57BL/6 mice were labeled with 5 μM eFluor 450 cell proliferation dye (eBioscience) and were stimulated with anti-CD3 (1 μg/ml). For preparation of pre-activated CD8+ T cells, splenocytes from C57BL/6 mice were stimulated with anti-CD3 (1 μg/ml) for 48 h, followed by gradual 2–3-fold media expansion with IL-2 (10 ng/ml; Peprotech) for 5 days. Live cells were collected by density gradient separation (Ficoll, GE Healthcare) and then re-stimulated with plate-bound anti-CD3 (1 μg/ml) and soluble anti-CD28 (2 μg/ml) in the presence of GolgiPlug (BD Biosciences) overnight. For short term stimulation, CD4+ T cells were harvested from 5C.C7 mice and purified by negative selection with CD4+ MACS cell isolation protocol (Miltenyi Biotec).

### Immunoblot Analysis

CD4+ T cells from 5C.C7 CD4+ transgenic mice were isolated with MACS. Samples were flash frozen and lysed in RIPA lysis buffer with protease and phosphatase inhibitor cocktails. Proteins were detected by ECL Plus substrate (GE Healthcare). All images were obtained using UVP Biospectrum500 Imaging System.

### Transplantation

Full-thickness BALB/c or FVB-Tg(CAG-luc,-GFP)L2G85Chco/J trunk skin grafts (1 cm^2^) were transplanted onto the flank of C57BL/6, FVB/N, or B6(Cg)-Tyrc-2J/J recipient mice, sutured with 6.0 Nylon and secured with dry gauze and a bandage for 7 days as previously described (33). Grafts were clinically observed every day thereafter and considered rejected when ≥90% of the graft tissue became necrotic.

### Heterotopic Heart Transplantation

BALB/c mice served as heart donors and C57BL/6 mice serve as allograft recipients.

Either abdominal or cervical heterotopic heart transplantation was performed as previously described (34, 35). Functionality of the transplanted heart was monitored daily by palpation was scored from 0 (no palpable heart beat) to 4 (strong, fast, rhythmic) according as previously described (36). Clinical rejection was defined by cessation of palpable heartbeats and confirmed by autopsy. Loss of graft function within 48 h of transplantation was considered as a technical failure, and animals in which this occurred were omitted from the analysis.

### Treatment Protocols

CTLA4Ig (Abatacept; Bristol-Myers) was administered at a dose 0.5 mg on days 0, 2, 4, 6 after transplantation. Triple metabolic therapy consisted of 2-DG, metformin and DON. 2DG 500 mg/kg and metformin 150 mg/kg were administered every day. DON 1.6 mg/kg was administered every other day. 2DG was purchased from Carbosynth. Metformin and DON were purchased from Sigma-Aldrich and Bachem.

For all *in vivo* experiments, CTLA4Ig and individual metabolic inhibitors were dissolved in PBS and administrated intraperitoneally (i.p.).

### *In vivo* Bioluminescence Imaging of Mice

Mice were anesthetized with 2% isoflurane and placed in a light-tight chamber. A photographic (gray-scale) reference image was obtained at 5 min after D-luciferin (Sigma) injection (150 mg/kg i.p.); bioluminescent images were collected immediately thereafter. Bioluminescence of the mice was detected via the IVIS Imaging System 200 Series. The region of interest from displayed images was designated and quantified as total flux (photons/sec) using Living Image 2.50 software (Xenogen).

### Donor Specific Antibody Assay

Donor Balb/c splenocytes (1 × 10^6^ cells) were incubated with diluted (1:50) serum from transplanted, sensitized or naïve recipients. After two washes, cells were stained with anti-B220, anti-CD3 and anti-IgG antibodies. Mean fluorescence intensity on the B220-negative cells were measured by flow cytometry.

### Flow Cytometry and Intracellular Cytokine Staining

Flow cytometry data were acquired with FACSCelesta (BD Biosciences) and were analyzed with FlowJo7.6 software (TreeStar). For intracellular staining, cells were stimulated at 37°C for 4 h in the presence of monensin (GolgiStop; BD Biosciences), phorbol 12-myristate 13- acetate (PMA; Sigma), and ionomycin (Sigma). Cells were surface stained and underwent fixation/permeabilization with either a Cytofix/Cytoperm kit (BD Biosciences) or a Fixation/Permeabilization kit (eBioscience), followed by staining for intracellular cytokines. Gates were determined appropriately using un-stimulated control cells. Voltages were determined from unstained controls.

### Statistical Analysis

Prism software version 7.0 (GraphPad Software) was used for statistical analyses, including one-way ANOVA non-parametric Kruskal–Wallis test, two-way ANOVA and log-rank analysis. A *p*-value less than 0.05 was considered statistically significant.

## Results

### CTLA4Ig and Metabolic Inhibitors Differentially Affect T Cell Activation

**Figure 1:**
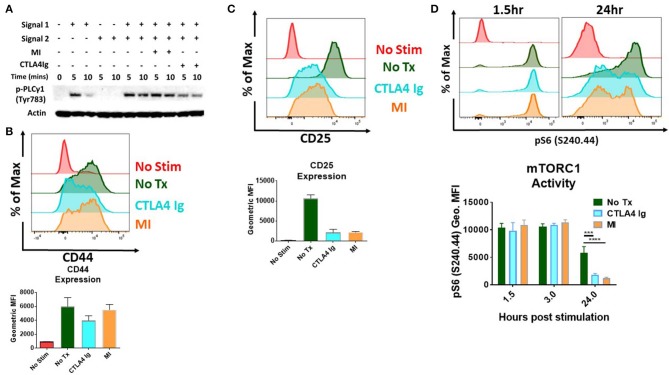
Effect of metabolic inhibitors (MI) and CTLA4Ig on T cell activation and mTORC1 activity **(A)** Immunoblot analysis of phospho-PLC gamma (Tyr 783) activity in naive CD4+ T cells stimulated with anti-CD3 (Signal 1) and/or anti-CD28 (Signal 2) in the presence of CTLA4Ig (50 μg/ml) or MI (2DG 0.6 mM, metformin 1 mM, DON 5 μM) for 5 and 10 min. **(B–D)** Splenocytes from naïve WT C57BL/6 mice were stimulated with anti-CD3 (1 μg/ml) and cultured in the presence of CTLA4Ig or MI as in **(A)**. **(B)** Expression of the activation marker CD44 on viable CD8+ T cells at 24 h under CTLA4Ig or MI treatment. **(C)** Expression of the activation marker and high affinity IL-2 Receptor CD25 **(D)** T cell activation induced mTORC1 activity determined by the phosphorylation state of the S6 ribosomal protein of viable CD8+ T cells at 1.5, 3, and 24 h. ***p < 0.001 ****p < 0.0001 (one-way ANOVA nonparametric Kruskal–Wallis test) Data are representative of at least three experiments **(A–D)**, (**B–D**, *n* = 4 biological replicates).

Initial studies were performed to examine the effects of CD28/B7 costimulation blockade and metabolic inhibitors (MI) on T cell activation. To this end, we stimulated T cells and performed immunoblot analysis and measured activation parameters, proliferation and cytokine production in the presence of CTLA4Ig or 2DG+metformin+DON (triple metabolic inhibitor (MI) therapy). As expected, T cell activation was impaired by CTLA4Ig, as indicated by reduced phosphorylation of PLC gamma upon early T cell signaling and also reduced expression of the activation marker CD44 (5 and 10 min) (Figures 1A,B). That is, blocking costimulation with CTLA4Ig inhibited TCR-induced signaling necessary for full T cell activation. Alternatively, blocking metabolism with the triple metabolic therapy only minimally affected proximal TCR-induced signaling and expression of CD44. In contrast, CD25 expression, which is induced by both TCR signaling and IL-2 signaling was equally inhibited by MI and costimulatory blockade (Figure 1C). In light of the important role of mTORC1 signaling in promoting T cell activation, differentiation and function (15), we also examined the effects of costimulatory blockade and MI on mTOR activation. As seen in Figure 1D, that while early mTORC1 signaling was not affected, both costimulatory blockade and MI led to a marked decrease in mTOR signaling by 24 h.

### CTLA4Ig and Metabolic Inhibitors Differentially Affect T Cell Proliferation, Activation Induced Cell Death and Function

**Figure 2:**
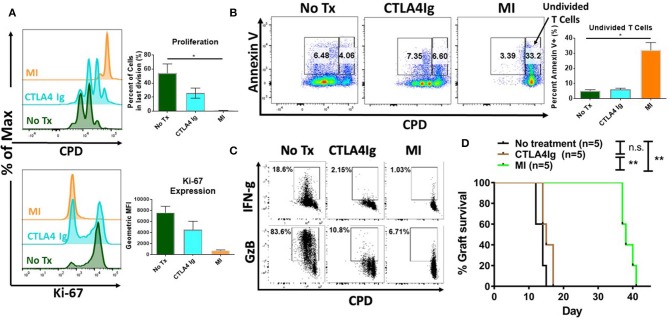
Effect of metabolic inhibitors (MI) and CTLA4Ig on T cell activation and BALB/c (H2d) to C57BL/6 (H2b) complete MHC-mismatched full thickness skin transplantation. Cell proliferation dye eFluor 450 (CPD) labeled splenocytes from naïve WT C57BL/6 mice were stimulated with anti-CD3 (1 μg/ml) and cultured in the presence of CTLA4Ig (50 μg/ml) or MI (2DG 0.6 mM, metformin 1 mM, DON 5 μM). **(A)** Proliferation and Ki-67 expression of viable CD8+ T cells at 48 h. **(B)** Percentage of Annexin V+ staining vs. proliferation in undivided cells (right gate) vs. proliferative cells (left gate) shown. **(C)** At 36 h after activation, brefeldin A was added to each culture for 12 h and IFN-gamma (IFN-g) and Granzyme B (GzB) production was compared to CPD dilution in CD8+ T cells. **(D)** Balb/c to C57BL/6 complete MHC-mismatched full thickness skin transplantation. Therapy was started from the day of transplantation (day 0) until complete graft rejection. No treatment MST = 14 days; CTLA4Ig MST = 17 days; MI MST = 38 days. n.s., not significant, *p < 0.05, **p < 0.01(log-rank analysis) (one-way ANOVA non-parametric Kruskal–Wallis test) Data are representative of at least three experiments **(A–C)**, (**A,B**, *n* = 4 biological replicates) and two experiments **(D)**.

Next, we sought to determine the potential differential effects of costimulatory blockade and MI on T cell proliferation and activation induced cell death. As seen in Figure 2A, unlike CTLA4Ig, MI strongly inhibited proliferation based on cell proliferation dilution (CPD) as cells were not able to fully enter cell cycle based on the expression of the proliferation marker, Ki-67. Having demonstrated that MI more robustly inhibited proliferation compared to costimulatory blockade, we next examined the effect of these regimens on activation induced cell death. As seen in Figure 2B, MI treatment resulted in markedly enhanced apoptosis of undivided T cells as determined by Annexin V positive staining. Thus, when compared to costimulatory blockade, MI inhibits clonal expansion by both blocking proliferation and promoting activation induced cell death. Next, we examined the effect of costimulatory blockade and MI on T cell function. We also observed both IFN-g and Granzyme B (GzB) production were markedly inhibited by both CLTLA4Ig and MI (Figure 2C). Finally, to test the ability of CTLA4Ig costimulation blockade and MI in suppressing alloimmune response, we performed BALB/c to C57BL/6 full thickness skin transplants and treated the recipients from the day of surgery with CTLA4Ig or 2DG+metformin+DON. As previously shown (31), blocking glycolysis, OXPHOS and glutamine metabolism significantly increased skin graft median survival time (MST) 38 days) compared to those that received CTLA4Ig (MST 15 days) or no treatment (MST 14 days) (Figure 2D). These findings suggest that CTLA4Ig and metabolic therapy have distinct immunosuppressive effects and that the ability of MI to limit clonal expansion by both preventing proliferation and enhancing activation induced cell death is associated with a more robust ability to prevent skin allograft rejection.

### Metabolic Inhibitors Have Increased Efficacy During Acute Rejection

**Figure 3:**
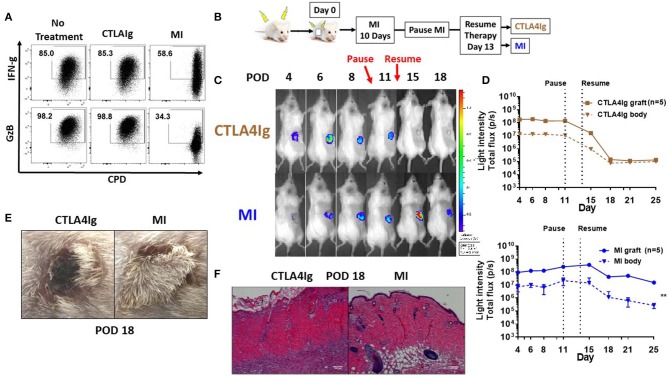
Effect of metabolic inhibitors and CTLA4Ig on pre-activated CD8+ T cells and a model of acute skin allograft rejection**. (A)** Previously activated and resting *in vitro* T cells were re-stimulated for 48 h with plate-bound anti-CD3 (1 μg/ml) and soluble anti-CD28 (2 μg/ml) in the presence of CTLA4Ig and MI as in Figure 1C. At 36 h, brefeldin A was added to each culture and IFN-gamma (FN-g) and Granzyme B (GzB) production was compared to eFluor 450 dilution in CD8+ T cells. **(B)** Schematic of transplant and treatment model. FVB-Tg(CAG-luc, -GFP)L2G85Chco/J (H2q) to B6(Cg)-Tyrc-2J/J (H2b) complete MHC-mismatched full thickness skin transplantation. MI therapy was started from the day of transplantation (day 0) until POD 10 and stopped for 3 days to induce acute rejection. On POD 14, treatment was resumed with either CTLA4Ig or MI. MI: metabolic inhibitors: 2DG+ metformin + DON. 2DG, 500 mg/kg once daily; metformin 150 mg/kg once daily; DON 1.6 mg/kg once every other day; CTLA4Ig 0.5 mg every other day starting on day 14. **(C)** Representative images from a single mouse for each of the indicated time points. **(D)** Skin graft viability measured by luminescent light intensity (photons/sec). **(E)** Skin graft appearance on POD 18. **(F)** Hematoxylin and eosin stain (X200) of skin grafts on POD 18. **p < 0.01 (2-way ANOVA). Data are representative of three experiments **(A)** and two experiments **(B–D)**.

Clinical trials with CTLA4Ig costimulation blockade have shown higher incidence of acute rejection during the early post-transplant period (21, 22). In light of the differences we observed comparing CTLA4Ig and MI in terms of inhibition of proliferation and activation induced cell death, we wanted to compare these two modalities in terms of their ability to inhibit acute rejection. First, we compared the effects of CTLA4Ig or MI on pre-activated T cells. We activated splenocytes with anti-CD3 for 48 h, expanded in media containing IL-2 for 5 days and then re-stimulated with plate bound anti-CD3 and soluble anti-CD28 for additional 48 h in the presence of CTLA4Ig or the metabolic inhibitors. Notably, CTLA4Ig had minimal effect on either proliferation or cytokine production, whereas metabolic therapy still had a partial effect on inhibiting previously activated T cell responses (Figure 3A). Next, we sought to compare the effects of CTLA4Ig/MI in a transplant model of acute rejection. To this end, we transplanted skin grafts from luciferase+ mice, FVB-Tg(CAG-luc,-GFP)L2G85Chco/J (H2q), to B6(Cg)-Tyrc-2J/J (H2b) recipients and monitored graft viability by tracking the intensity of their bioluminescent signal. Luciferase+ mice have been widely used as donors for monitoring engraftment of transplanted heart, ovary, hepatocyte and islet (37–40). In skin transplants, allogenic grafts lose viability and decrease bioluminescent signal early after day 6 post-surgery. By Day 12, they are rejected completely. In contrast, syngenic grafts maintain the bioluminescent signal over time (Supplementary Figure 1). Given that MI has been shown to be more effective than other conventional immunosuppressive regimens such as cyclosporine or rapamycin in prolonging allograft survival (33), for this acute rejection model, we first treated all the recipients with MI for 10 days to minimize the initial inflammation and stabilize the graft. On Day 10, we paused treatment to induce an alloimmune response and acute rejection, and we resumed treatment after 3 days either with CTLA4Ig or MI (Figure 3B). Under CTLA4Ig treatment, total graft loss, as evidenced by bioluminescence and histology, occurred on day 18 (Figures 3C–F), which is equivalent to the normal rejection time of non-treated allogeneic grafts (Supplementary Figures 1B,C). With MI, however, graft viability persisted at least for another 7 days. Thus, consistent with previous reports which suggested that memory cells may be resistant to CD28/B7 costimulation blockade (41, 42) MI appeared to be superior to CTLA4Ig in terms of treating acute rejection.

### CTLA4Ig and MI Have Synergistic Effects on Inhibiting Acute and Memory T Cell Responses

**Figure 4:**
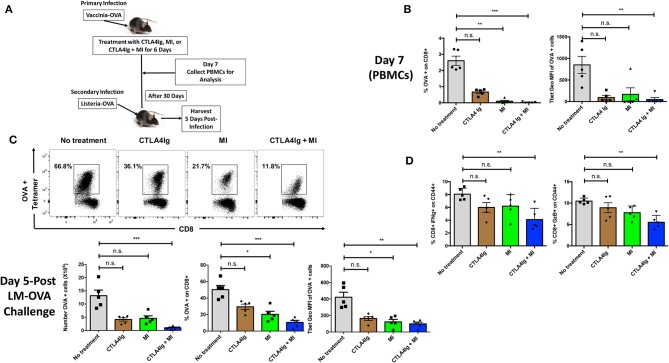
Effect of combining MI and CTLA4Ig on a robust model of T cell effector response. **(A)** Schematic of infection model with treatment. WT C57BL/6 mice were infected with OVA-expressing Vaccinia virus and treated with MI (2DG + metformin + DON; 2DG, 500 mg/kg once daily; metformin 150 mg/kg once daily; DON 1.6 mg/kg once every other day) or MI + CTLA4Ig (CTLA4Ig 0.5 mg once every other day) for 6 days. Peripheral blood was collected on Day 7 to analyze antigen-specific CD8+ OVA tetramer+ T cells. 30 days after the primary infection, the same mice were infected with OVA-expressing Listeria monocytogenes for memory T cell recall response. Five days following the secondary infection, splenocytes were isolated to analyze memory response. **(B)** Frequency and T-bet expression (geometric MFI) of tetramer+ CD8+ T cells on Day 7. **(C)** Total number, frequency and T-bet expression of OVA+ CD8+ T cells after re-challenge. **(D)** Percentage of IFN-gamma (IFN-g) + and Granzyme B (GzB) + CD8+ CD44+ cells after 5 h *ex vivo* re-stimulation with OVA peptide. n.s., not significant, *p < 0.05, **p < 0.01, ***p < 0.001 (one-way ANOVA non-parametric Kruskal–Wallis test). Data are representative of two experiments.

Having demonstrated distinct differences between the ability of MI and CTLA4Ig to inhibit T cell function, we next sought to test the ability of *combination* therapy to inhibit antigen–specific T cell responses in a robust infection model. To this end, C57BL/6 mice were infected with vaccinia-OVA and treated with CTLA4Ig, MI or CTLA4Ig+MI for 6 days. After 30 days of the primary infection, a secondary infection with listeria-OVA was performed to induce antigen-specific memory recall. This model enables us to specifically track and examine the effect of MI + CTLA4Ig on robustly activated antigen specific T cells *in vivo*. To this end, the antigen-specific CD8+ T cell response was interrogated by Class I OVA+ tetramer staining. The acute response was analyzed in peripheral blood on Day 7 and memory re-call was examined in splenocytes on Day 35, 5 Days after the secondary infection (Figure 4A). Consistent with the ability of MI to robustly inhibit clonal expansion, treatment with MI inhibited the expansion of tetramer+ CD8+ T cells more significantly than CTLA4Ig (Figure 4B). However, combination with CTLA4Ig resulted in a more profound inhibitory effect and furthermore, a stronger decrease in T-bet expression of those T cells (Figure 4B). In addition, we monitored cell proliferation of adoptively transferred CD8+ OTI T cells with acute drug therapy in response to vaccinia-OVA infection. Similar to our *in vitro* findings (Figure 2), MI or CTLA4Ig+MI therapy significantly decreased proliferation of transferred CD8+ T cells (Supplementary Figure 2A). Also, we observed increased cell death in the undivided T cell population with MI or CTLA4Ig+MI therapy unlike proliferative T cells that typically die during the course of infection seen in the no treatment group (Supplementary Figure 2B). Following secondary infection (re-challenge), CTLA4Ig combined with MI not only markedly suppressed memory recall (Figure 4C), but also led to a stronger inhibition of IFN-g or Granzyme B secreting CD8+ T cells (Figure 4D). Together, these results show that combination therapy of CTLA4Ig with MI had the most robust effect on CD8+ T cell expansion, both during the initial antigen encounter and during memory recall.

### Combined CTLA4Ig and MI Prevent Allograft Rejection in Skin Transplantation and Promote Graft Acceptance Upon Stopping Therapy in Heart Transplantation

**Figure 5:**
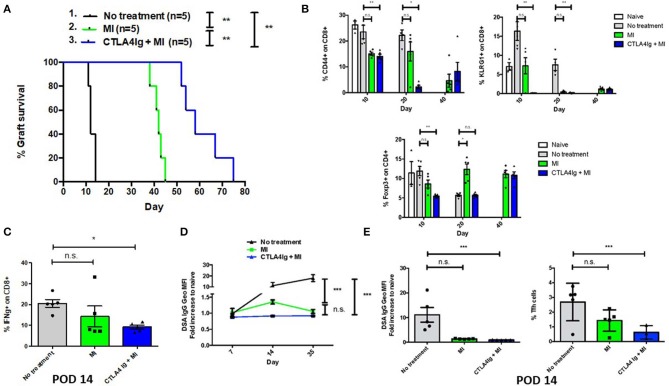
Effect of combining metabolic inhibitors and CTLA4Ig on promoting allograft tolerance in BALB/c (H2d) to C57BL/6 (H2b) complete MHC-mismatched full thickness skin transplantation. The dosage of all drugs was the same as described in Figure 2D. Therapy was started from the day of transplantation (day 0) until complete graft rejection. **(A)** Skin allograft survival comparing MI alone with MI + CTLA4Ig. No treatment MST = 12 days; MI MST = 42 days; CTLA4Ig + MI MST = 58 days. **(B)** Dynamics of levels of CD44+ and KLRG1+ activated effector CD8+ T cells and Foxp3+ CD4+ regulatory T cells in peripheral blood over time. **(C)** Percentage of IFN-gamma (IFN-g) + CD8+ cells after 4 h *ex vivo* re-stimulation with PMA and ionomycin **(D)** Levels of donor-specific IgG antibodies (DSA, presented as fold increased relative to naive mice) during early and late post-tranplant period. **(E)** Levels of DSA and frequency of follicular helper T (Tfh) cells (CD4+ ICOS+ PD1+ Bcl6+) in draining LNs on POD 14. n.s., not significant, *p < 0.05, **p < 0.01, ***p < 0.001 (log-rank analysis and one-way ANOVA non-parametric Kruskal–Wallis test). Data are representative of two experiments.

Having demonstrated the effect of CTLA4Ig and MI in inhibiting acute and memory responses in virus infection, we next examined the ability of the combination therapy in improving Balb/c to C57BL/6 skin and heart allograft survival. Not surprisingly, CTLA4Ig+MI significantly prolonged skin graft survival (Figure 5A). Levels of peripherally circulating T cells were analyzed periodically and showed decreased frequency of activated (CD44+) and terminally differentiated (KLRG1+) CD8+T cells during early time points (POD 10 and 20) in the combination group. Regulatory T cells were decreased initially, but then recovered over time (POD 40) (Figure 5B). Additionally, CTLA4Ig+MI strongly inhibited IFN gamma production of CD8+ T cells upon re-challenge *ex vivo* (Figure 5C) and donor specific antibodies (DSA), were steadily low over time (Figure 5D). This correlated with decreased levels of follicular T helper cells (Figure 5E), which is in line with data showing that CTLA4Ig interferes with T cell- B cell help and decrease antibody-mediated rejection (43, 44). Thus, in the robust model of skin allograft rejection, combining MI therapy with CTLA4Ig led to prolonged graft survival.

**Figure 6:**
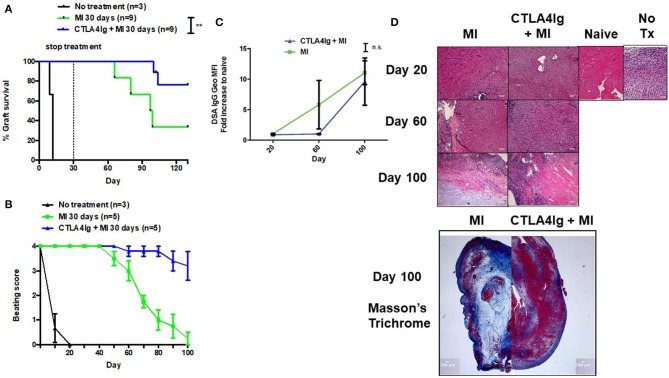
Effect of combining short-term metabolic therapy and CTLA4Ig on BALB/c (H2d) to C57BL/6 (H2b) complete MHC-mismatched heterotropic heart transplantation. The dosage of all drugs was the same as described in Figure 2D. Metabolic therapy was started from the day of transplantation (day 0) until POD 30. **(A)** Heart allograft survival. **(B)** Clinical assessment of the heart grafts by cervical or abdominal palpation (grade 0–4). **(C)** Levels of DSA over time. **(D)** Hematoxylin and eosin stain (X200) of heart grafts at POD 20, 60, and 100. Masson's trichrome stain (X20) of heart grafts at POD 100. n.s., not significant, **p < 0.01 (log-rank analysis and one-way ANOVA non-parametric Kruskal–Wallis test). Data pooled from two experiments.

Finally, we tested the CTLA4Ig+MI regimen in heart transplants. Previously, we have shown that continuous therapy with MI alone could achieve more than 100-day graft survival (31). However, stopping treatment resulted in rejection of the hearts 80 days later (data not shown). Therefore, we investigated whether costimulatory blockade could be able to improve the efficacy of short-term anti-metabolic therapy. Consistent with previous preliminary studies, treatment with only 30 days of MI resulted in rejection of the grafts ~60 days later (Figure 6A). In contrast, adding CTLA4Ig the first week after transplant could achieve 100% 100-day graft survival and near 80% 130-day graft survival, which represents 100 days without treatment (Figure 6A). Nevertheless, clinical assessment of the beating scores showed a decline in the beating quality of the transplanted hearts, although to a lesser extent than the MI alone group (Figure 6B). The addition of CTLA4Ig also resulted in an initial decrease in DSA production that then increased after stopping therapy (Figure 6C). Interestingly, histologic examination of the hearts on POD 100 revealed more viable heart tissue with less necrosis and fibrosis in the setting of equivalent lymphocytic infiltration between the groups (Figure 6D).

## Discussion

In a previous study (31), we demonstrated that metabolic inhibition therapy (MI) in the form of 2-DG (a glucose analog), DON (a glutamine antagonist) and metformin (a diabetes drug) could prevent allograft rejection. However, discontinuation of treatment led to graft rejection. Thus, the overall goal of this study was determine if the addition of CTLA4Ig might promote long-term graft acceptance in the absence of long-term immunosuppressive therapy. To this end, in this current study we have (**i)** Demonstrated distinct immunologic effects between MI and CTLA4Ig therapy (**ii)** The ability of MI therapy to treat acute graft rejection (**iii)** The synergistic ability of MI + CTLA4Ig to prevent skin allograft rejection (**iv)** The ability of short term MI + CTLA4Ig therapy to promote long-term heart allograft acceptance. While our studies specifically focused on the combination of MI + CTLA4Ig, we believe that these results provide the rationale for employing metabolic therapy as a platform for a potential wide variety of tolerance-inducing regimens.

CTLA4Ig costimulatory blockade is currently widely used in clinical organ transplantation (45–48). Recent long-term clinical trials in kidney transplant patients have shown improved graft function, better cardiovascular/metabolic risk profile and similar patient and graft survival when compared to CNIs. However, these trials also noticed higher rates of acute rejection within the first months post-transplantation (21, 22). The acute rejection episodes were not typically associated with DSA (49). T cell mediated acute cellular rejection may be alternatively involved and our study demonstrated that anti-metabolic therapy had a more profound effect on suppressing effector T cell function during acute rejection or acute viral infection. Indeed, similar to other immunosuppressive regimens the profound ability of metabolic therapy to block anti-allograft clonal expansion would also suppress clonal expansion in response to concomitant infections. Nonetheless, metabolic therapy has a number of advantages over treatment regimens that include CNIs. First, a major problem of conventional immunosuppression with CNI's is Post-transplant lymphoproliferative disorder (PTLD) as well as the reactivation of herpes viruses such as CMV. Metabolic therapy on the other hand directly inhibits herpes virus activation as well as inhibits cancer cell growth (50, 51). Second, in as much as CNI's inhibit tolerance induction, transplant patients require life-long immunosuppression. Metabolic therapy promotes tolerance thus potentially mitigating the need for prolonged immunosuppression and hence susceptibility to infection. Finally, from a clinical perspective CNIs (and steroids) are associated with multiple adverse events including hyperglycemia, accelerated atherosclerosis, gastro intestinal bleeding, neurotoxicity, and nephrotoxicity all of which are avoided and in some cases (for example metformin and hyperglycemia) improved with metabolic therapy.

Further, while CTLA4Ig was associated with decreased TCR-induced activation, MI was associated with a marked decrease in clonal expansion. This decrease was mechanistically secondary to both a decrease in proliferation and an increased in activation induced cell death. Along these lines the initial antigen-specific T-cell precursor frequency has been shown to be an important factor in determining the effectiveness of CTLA-4Ig in a murine model of transplantation suggesting that patients with an initially high precursor frequency of alloreactive T cells (poor major histocompatibility complex donor and recipient matching) might also be particularly refractory to treatment with CTLA4Ig costimulatory blockade (52). Our data suggest that the addition of MI therapy can help overcome this hurdle. Furthermore, we observed that combination therapy led to further decreased DSA formation by decreasing follicular T helper cells and abrogating T cell- B cell help. Likewise, MI therapy led to a decrease in mTORC1 activation that could further contribute to the ability of MI + CTLA4Ig to promote long-term graft acceptance.

In as much as we observed graft acceptance in the absence of continued immunosuppression, our results suggest that MI + CTLA4Ig can induce a state of “functional” tolerance. That said, it should be noted that upon stopping therapy we observed an increase in DSA and that in spite of having less fibrosis and more functional heart tissue in the MI + CTLA4Ig treated hearts when compared to the MI treated hearts, we did observe equivalent lymphocytic infiltration POD 100. Also, secondary skin transplants to those recipients in the combination group that survived more than 150 days showed normal rejection of the skin grafts (data not shown), indicating that robust tolerance was not induced. However, neither the heart graft of those recipients rejected after the secondary re-challenge nor the beating quality further decreased. To this end, while our work supports the concept of MI therapy as a platform for inducing tolerance, the details of robustly achieving this goal remain to be determined.

## Data Availability Statement

The datasets generated for this study are available on request to the corresponding author.

## Ethics Statement

All animal procedures were in accordance with the guidelines of the Institutional Animal Care and Use Committee (IACUC) at Johns Hopkins University.

## Author Contributions

GB and JP designed research. C-HC, C-FL, BO, GF, and CP performed research. C-HC analyzed data. C-HC and JP wrote the paper.

### Conflict of Interest

JP has equity in Dracen, Sitryx (<5%) and Corvus (<5%); has consulted for Dracen, as well as for Sitryx, Corvus, Aeonian, Sigma and Quadriga; has received sponsored research money unrelated to the current paper from Abbvie, Quadriga, Dracen, Bluebird and Bristol- Myers Squibb in the last year; and has patents licensed by Dracen. The remaining authors declare that the research was conducted in the absence of any commercial or financial relationships that could be construed as a potential conflict of interest. The reviewer CB and handling Editor declared their shared affiliation at the time of review.

## Supplementary Material

Supplemental Figure 1 *In vivo* bioluminescence to assess changes of skin allograft viability during the course of alloimmune rejection. FVB-Tg(CAG-luc,-GFP)L2G85Chco/J (H2q) to FVB/N (H2b) and B6(Cg)-Tyrc-2J/J (H2b) syngenic and allogenic full thickness skin transplantation. **(A)** Representative images from a single mouse for each of the indicated time points. **(B)** Skin graft viability measured by luminescent light intensity (photons/sec). **(C)** Skin graft appearance on POD 12. Data are representative of two experiments.

Supplemental Figure 2 Effect of metabolic inhibitors (MI) and CTLA4Ig on T proliferation and cell viability in response to viral infection. WT C57BL/6 mice were infected with OVA-expressing Vaccinia virus prior to receiving different congenic marked ef450-labeled CD8+ OTI T cells. Mice were then treated with MI (2DG + metformin + DON; 2DG, 500 mg/kg once daily; metformin 150 mg/kg once daily; DON 1.6 mg/kg once daily) or MI + CTLA4Ig (CTLA4Ig 0.5 mg once) for 2 days. On Day 2, spleens were harvested to analyze CPD dilution and cell viability of donor CD8+ T cells. **(A)** Percent proliferation based on CPD dilution of donor CD8+ T cells. **(B)** Percent death seen in undivided cells vs. divided cells ^*^*p* < 0.05, ^**^*p* < 0.01, ^****^*p* < 0.0001 *n* = 5–8 per group (one-way ANOVA non-parametric Kruskal–Wallis test). Data are representative of three independent experiments.
