# Supplementary figures and images for: Targeting Metabolism as a Platform for Inducing Allograft Tolerance in the Absence of Long-Term Immunosuppression
Source: Front Immunol. 2020 Apr 9;11:572. doi: 10.3389/fimmu.2020.00572 (PMC7161684; doi:10.3389/fimmu.2020.00572)

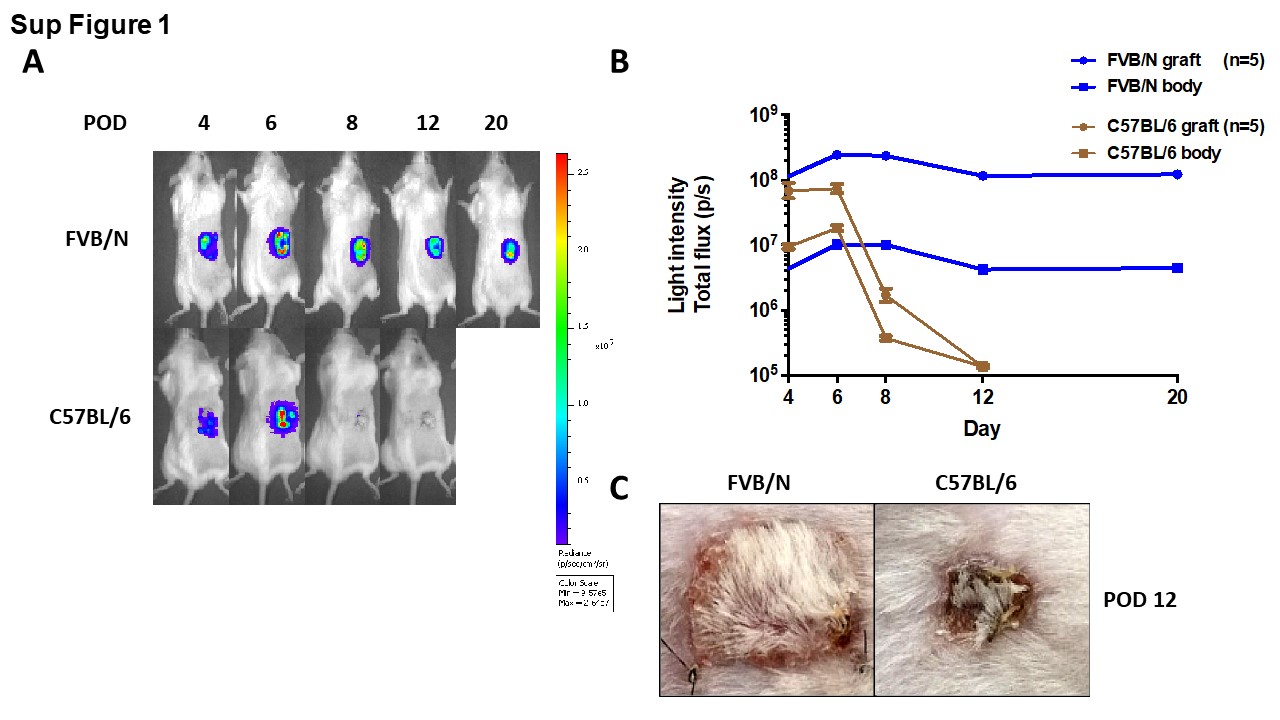

Supplement: Supplemental Figure 1 — In vivo bioluminescence to assess changes of skin allograft viability during the course of alloimmune rejection. FVB-Tg(CAG-luc,-GFP)L2G85Chco/J (H2q) to FVB/N (H2b) and B6(Cg)-Tyrc-2J/J (H2b) syngenic and allogenic full thickness skin transplantation. (A) Representative images from a single mouse for each of the indicated time points. (B) Skin graft viability measured by luminescent light intensity (photons/sec). (C) Skin graft appearance on POD 12. Data are representative of two experiments. [file Image_1.jpeg]

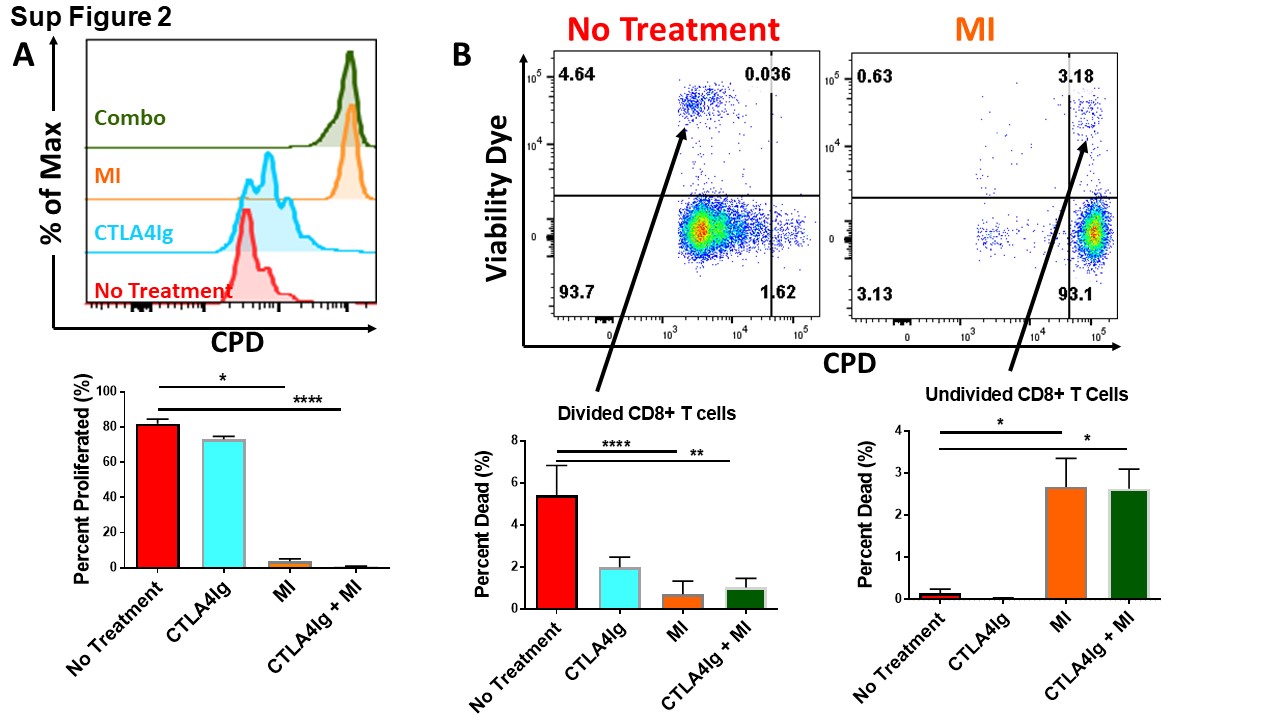

Supplement: Supplemental Figure 2 — Effect of metabolic inhibitors (MI) and CTLA4Ig on T proliferation and cell viability in response to viral infection. WT C57BL/6 mice were infected with OVA-expressing Vaccinia virus prior to receiving different congenic marked ef450-labeled CD8+ OTI T cells. Mice were then treated with MI (2DG + metformin + DON; 2DG, 500 mg/kg once daily; metformin 150 mg/kg once daily; DON 1.6 mg/kg once daily) or MI + CTLA4Ig (CTLA4Ig 0.5 mg once) for 2 days. On Day 2, spleens were harvested to analyze CPD dilution and cell viability of donor CD8+ T cells. (A) Percent proliferation based on CPD dilution of donor CD8+ T cells. (B) Percent death seen in undivided cells vs. divided cells *p < 0.05, **p < 0.01, ****p < 0.0001 n = 5–8 per group (one-way ANOVA non-parametric Kruskal–Wallis test). Data are representative of three independent experiments. [file Image_2.jpeg]
